# Androgen Receptor Signaling in the Development of Castration-Resistant Prostate Cancer

**DOI:** 10.3389/fonc.2019.00858

**Published:** 2019-09-04

**Authors:** Qin Feng, Bin He

**Affiliations:** ^1^Department of Biology and Biochemistry, Center for Nuclear Receptors and Cell Signaling, University of Houston, Houston, TX, United States; ^2^Departments of Surgery and Urology, Immunobiology & Transplant Science Center, Houston Methodist Cancer Center, Houston Methodist Research Institute, Houston Methodist Hospital, Houston, TX, United States; ^3^Department of Medicine-Cancer Biology, Weill Cornell Medicine, Cornell University, New York, NY, United States

**Keywords:** prostate cancer (PCa), androgen receptor (AR), androgen deprivation therapy (ADT), castration-resistant prostate cancer (CRPC), small-cell prostate cancer (SCPC), antiandrogen

## Abstract

Most prostate cancers are androgen-sensitive malignancies whose growths depend on the transcriptional activity of the androgen receptor (AR). In the 1940s, Charles Huggins demonstrated that the surgical removal of testes in men can result in a dramatic improvement in symptoms and can induce prostate cancer regression. Since then, androgen deprivation therapies have been the standard first-line treatment for advanced prostate cancer, including: surgical castration, medical castration, antiandrogens, and androgen biosynthesis inhibitors. These therapies relieve symptoms, reduce tumor burden, and prolong patient survival, while having relatively modest side effects. Unfortunately, hormone deprivation therapy rarely cures the cancer itself. Prostate cancer almost always recurs, resulting in deadly castration-resistant prostate cancer. The underlying escape mechanisms include androgen receptor gene/enhancer amplification, androgen receptor mutations, androgen receptor variants, coactivator overexpression, intratumoral *de novo* androgen synthesis, etc. Whereas, the majority of the castration-resistant prostate cancers continuously rely on the androgen axis, a subset of recurrent cancers have completely lost androgen receptor expression, undergone divergent clonal evolution or de-differentiation, and become truly androgen receptor-independent small-cell prostate cancers. There is an urgent need for the development of novel targeted and immune therapies for this subtype of prostate cancer, when more deadly small-cell prostate cancers are induced by thorough androgen deprivation and androgen receptor ablation.

## Androgens and the Androgen Receptor in the Prostate Gland

The prostate is a walnut sized male reproductive gland located between the bladder and the penis. It secretes the prostatic fluid that helps to nourish and transport sperm. Androgen signaling plays a pivotal role in the development and function of a normal prostate gland. There are two native androgens in humans, testosterone (T), and 5α-dihydrotestosterone (DHT). Testosterone is produced mainly in the testis, with a small amount being produced in the adrenal glands in men. Testosterone is converted to the more potent androgen dihydrotestosterone by the enzyme 5 alpha-reductase located in the prostate, skin, scalp, etc. Both testosterone and dihydrotestosterone can bind to a single nuclear receptor protein, the androgen receptor, which is an androgen-dependent transcriptional activator and a member of nuclear receptor superfamily.

**Figure 1:**
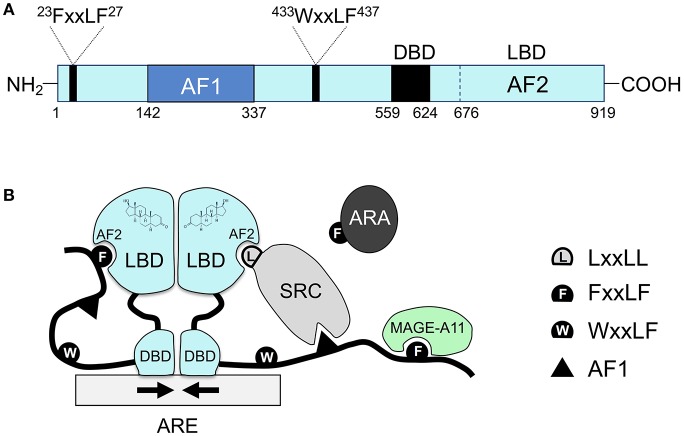
The unique molecular features of the androgen receptor and its coregulator recruitment. **(A)** The primary sequence of the androgen receptor contains several functional domains: NH_2_-terminal Activation Function 1 (AF1), the central DBD, the carboxyl-terminal LBD, and two AR-specific FxxLF and WxxLF motifs. **(B)** Schematic diagram of homodimeric androgen receptor bound to a palindromic androgen response element (ARE). Dimerization of the androgen receptor is mediated by both DBD and LBD. Shown in the diagram are FxxLF motif-mediated N/C interaction, recruitment of the SRC/p160 by AF1 and AF2, recruitment of FxxLF motif-containing ARA proteins by AF2, and recruitment of MAGE-A11 through the AR NH_2_-terminal extended FxxLF motif. Competition likely exists among different FxxLF, WxxLF, and LxxLF motifs for binding to the same AF2 site on AR LBD (1). SRC, steroid receptor coactivator; ARA, AR-associated protein; AF1, activation function 1; AF2, activation function 2, a hydrophobic cleft in the LBD; ARE, androgen response element; DBD, DNA binding domain; LBD, ligand binding domain.

Similar to other nuclear hormone receptors, the androgen receptor protein contains three main functional domains: the NH_2_-terminal unstructured transcriptional activation domain, the central DNA binding domain (DBD), and the carboxyl-terminal ligand binding domain (LBD) (Figure 1A). Between DBD and LBD, there is a flexible hinge region (amino acid 624–676), which harbors a bipartite nuclear localization signal (NLS). In the classical model, the androgen receptor binds to androgen response elements (AREs) as a homodimer, and dimerization is mediated by both DBD and LBD (2, 3) (Figure 1B). Whereas, other nuclear receptors recruit LxxLL motif-containing coactivators such as the steroid receptor coactivator (SRC)/p160 family coactivators through their ligand binding domains, the androgen receptor ligand binding domain preferentially engages in the FxxLF motif-mediated NH_2_-terminal and carboxyl-terminal (N/C) interaction (4–7) or recruits FxxLF motif-containing coregulators (5, 8) (Figure 1B). Nevertheless, the androgen receptor can still recruit the SRC/p160 family of coactivators mainly through its unstructured NH_2_-terminus and LBD (9). Moreover, the androgen receptor can also recruit an AR-specific MAGE-A11 coactivator through its extended NH_2_-terminal FxxLF motif (10).

In the absence of hormones, the androgen receptor is associated with heat shock proteins and located in the cytoplasm in an inactive conformation. Upon androgen binding, the androgen receptor quickly undergoes conformational change, nuclear translocation, recognition of androgen responsive elements in the genomic DNA, and recruitment of coactivator machineries, resulting in transcription of target genes, such as prostate-specific antigen (PSA) and transmembrane protease serine 2 (TMPRSS2).

Dihydrotestosterone is a significantly more potent androgen than testosterone both *in vitro* and *in vivo*. While this variance in potency was commonly attributed to their different binding affinities, dihydrotestosterone actually binds to the androgen receptor with similar or somewhat higher affinity compared with testosterone (11, 12). In contrast, these two androgens bind to the androgen receptor with very differing kinetics (11, 12). The rate of dissociation for dihydrotestosterone from the androgen receptor is about three to five times slower than testosterone (11, 12). Therefore, it is largely their binding kinetics, rather than affinity, which accounts for the differential androgenic activities of these two hormones. As we know, in the field of drug discovery, the notion that drug-receptor binding kinetics could be as important as affinity in determining drug efficacy is becoming more widely accepted (13). In further support of the importance of androgen binding kinetics, the unique androgen receptor inter-domain N/C interaction slows the rate of androgen dissociation without affecting androgen binding affinity and is required for optimal target gene transcription (4).

## Co-evolution of Androgen Deprivation Therapy (ADT) and Prostate Cancer

### Surgical and Medical Castration

Prostate cancer occurs in the prostate gland. It is the most commonly diagnosed non-skin cancer and the second leading cause of cancer death in men in the United States. Based on his finding that the growth of prostate glands in dogs depended on testosterone, Charles Huggins demonstrated that surgical removal of testes in men, which produces more than 90 percent of testosterone, can result in a dramatic improvement in symptoms and can induce regressions of prostate cancers at any site (14). Since then, androgen deprivation therapy has been the standard first-line treatment for advanced prostate cancer (15). In addition to surgical castration, gonadotropin-releasing hormone (GnRH) analogs such as leuprolide, goserelin, and buserelin can suppress gonadotropin secretion and thus block the production of testicular androgens. As a result of its cosmetic and psychological concerns, medical castration via GnRH analogs has been the mainstay treatment for advanced prostate cancer.

### First-Generation Antiandrogens

**Figure 2:**
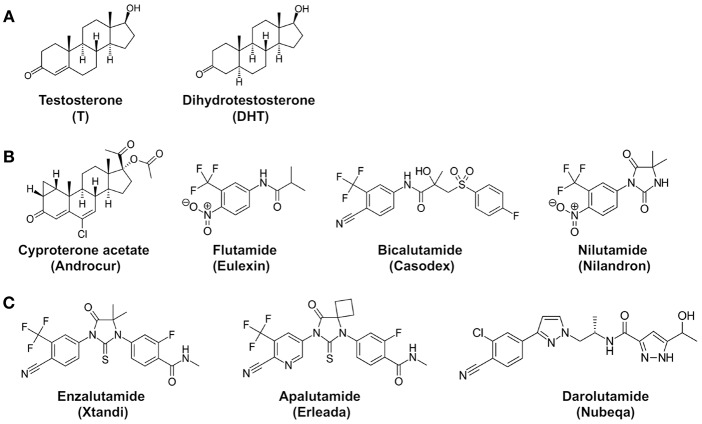
Chemical structures of androgens and antiandrogens. **(A)** Two main androgens, testosterone (T), and dihydrotestosterone (DHT). **(B)** First-generation antiandrogens, cyproterone acetate (Androcur), Flutamide (Eulexin), Bicalutamide (Casodex), Nilutamide (Nilandron). 2-hydroxyflutamide is the major active metabolite of flutamide in the body. **(C)** Representative second-generation antiandrogens, Enzalutamide (Xtandi), Apalutamide (Erleada), and Darolutamide (Nubeqa). Structures are adopted from Wikipedia.

Although surgical and medical castration can suppress testosterone production in the testes, the adrenal glands can still produce small amounts of androgens. To neutralize the activity of these residual androgens, antiandrogens were used to block androgen receptor signaling in prostate cancer cells (Figure 2). For example, cyproterone acetate (CPA), a synthetic steroid, was used as a prototypical antiandrogen (16). However, due to its relative ineffectiveness, CPA was replaced by more potent non-steroidal pure antiandrogens, such as Flutamide (Eulexin), bicalutamide (Casodex), and nilutamide (Nilandron). Unlike GnRH analogs, these antiandrogens do not prevent androgen production in the body. Instead, the antiandrogens bind to the androgen receptor with a relatively high affinity but lack the ability to activate transcriptional activity of the androgen receptor. Therefore, the antiandrogens function by competitively blocking testosterone and dihydrotestosterone from binding to the androgen receptor. For instance, flutamide and its active metabolite hydroxyflutamide bind to androgen receptors with a Ki of ~3,395 and ~134 nM, respectively (17). Bicalutamide is a more potent non-steroidal antiandrogen; its affinity for androgen receptors is two to four times more potent than hydroxyflutamide and nilutamide (18). Bicalutamide was thus modestly effective in prostate cancer patients who developed resistance after flutamide treatment (19). While effective on their own, antiandrogens are not usually used in monotherapy. Instead, they have proven to be used in conjunction with medical or surgical castration (20–22).

### “Androgen-Independence” to Castration-Resistance

The combination therapy of GnRH analogs and antiandrogens has promoted the survival of prostate cancer patients (21, 23). Unfortunately, most prostate cancers develop resistance to the combined androgen deprivation therapy after several years, becoming so-called “androgen-independent” prostate cancer. Surprisingly, it was found that, even after castration, the testosterone and dihydrotestosterone levels in locally recurrent prostate cancer tissues remain high enough to activate androgen receptors (24, 25). In support of this observation, the androgen receptor target gene PSA remains expressed in recurrent prostate cancer tissues, despite the castrate levels of androgens in serum (24, 25). Moreover, it has been reported that in the recurrent metastatic prostate cancers, intratumoral *de novo* androgen synthesis by overexpressed steroidogenic enzymes may contribute to elevated testosterone levels (26). Taken together, it becomes evident that recurrent cancers after medical or surgical castration are not truly androgen-independent (27), as they continuously depend on androgens and the androgen receptor to survive and grow. These recurrent cancers have been more appropriately classified as castration-resistant prostate cancer (28).

### Mechanisms of Castration Resistance

Subsequent studies have revealed multiple mechanisms which may contribute to the androgen receptor-dependence in castration-resistant prostate cancer. Firstly, increased androgen receptor expression can be caused by androgen receptor gene amplification (29–33) or androgen receptor enhancer amplification (34, 35). Secondly, increased expression of androgen receptor coactivators SRC1 and TIF2 stimulates androgen receptor activity in the presence of the weaker androgen androstenedione (36). The expression of the MAGE-A11 coactivator, which is recruited through androgen receptor NH_2_-terminal FxxLF motif, is increased in castration-resistant prostate cancer (37, 38). Thirdly, mutations in the androgen receptor ligand binding domain enable the androgen receptor to be activated by antiandrogens or other steroid hormones (39). For instance, the androgen receptor with the LNCaP mutation T877A can be activated by flutamide, estrogen, and progesterone (40, 41). The androgen receptor with L701H/T877A double mutations can be activated by glucocorticoids (42). Fourthly, constitutively active androgen receptor variants which lack ligand binding domains are another underlying mechanism of the castration resistance (43–45). Additionally, growth hormones such as epidermal growth factor (EGF) increase TIF2/GRIP1 coactivation of androgen receptor activity in recurrent cancer cells (46). Insulin-like growth factor-1 (IGF-1), keratinocyte growth factor (KGF), and EGF can all activate androgen receptor activity in the absence of androgens (47).

### Antiandrogen Withdrawal Syndrome

Interestingly, in some patients, when an antiandrogen is no longer working, simply stopping the antiandrogen treatment can stop cancer growth for a short period of time. This phenomenon is known as antiandrogen withdrawal syndrome. Decreases in PSA levels and/or clinical improvement after discontinuation of antiandrogens upon disease progression have been shown by flutamide, bicalutamide, and nilutamide withdrawal (48–50). One mechanism of antiandrogen withdrawal syndrome is acquired mutations in the androgen receptor ligand binding domain including mutation T877A and H874Y. Not surprisingly, these mutations have converted antiandrogens to androgen receptor agonists (51).

### Second-Generation Antiandrogens

To overcome castration resistance, more potent antiandrogens, known as second-generation antiandrogens, have been designed to achieve maximal androgen blockade (52). These second-generation antiandrogens include Enzalutamide (Xtandi), Apalutamide (Erleada), and Darolutamide (Nubeqa) (Figure 2). Enzalutamide and apalutamide are structurally similar to each other, having 5- to 8-fold higher binding affinities for androgen receptors in comparison to first-generation antiandrogens. Importantly, these antiandrogens function as pure antagonists for the androgen receptor in the presence of mutations such as T877A. Darolutamide is structurally distinct and shows 8- to 10-fold higher affinity for the androgen receptor than enzalutamide and apalutamide, and can inhibit the enzalutamide-resistant mutant androgen receptor (53). Therefore, darolutamide appears to be an even more potent second-generation antiandrogen. In addition to these three FDA-approved second-generation antiandrogens, other antiandrogens are also being developed. For instance, a potent AR inhibitor JNJ-73576253 (TRC253), developed by Janssen Pharmaceuticals, is a pan-inhibitor of AR, even in the presence of certain activating mutations, and is currently in Phase 1/2A clinical trial (54).

These more potent second-generation antiandrogens have been successful in prolonging the survival of men with castration-resistant prostate cancer. For instance, in men with metastatic castration-resistant prostate cancer after chemotherapy, enzalutamide produced an overall survival benefit of 4.8 months compared to the placebo (55). For patients with metastatic prostate cancer who have not received chemotherapy, enzalutamide also significantly increased progression-free survival and overall survival (56). Moreover, enzalutamide, apalutamide, and darolutamide all had significantly prolonged metastasis-free survival in men with high-risk non-metastatic castration-resistant prostate cancer (57–59). As shown by the latest phase III trials, both enzalutamide and apalutamide could significantly increase the progression-free survival and overall survival for men with metastatic hormone-sensitive prostate cancer (60–62).

In addition to the development of second-generation antiandrogens, Abiraterone (Zytiga) was developed as an irreversible steroid inhibitor of CYP17, a key enzyme in androgen synthesis. Abiraterone acetate inhibits the production of androgens in the testes, adrenal glands, and prostate tumors. In patients with metastatic castration-resistant and chemotherapy-resistant prostate cancer, Abiraterone produced an overall survival benefit of 3.9 months in comparison to the placebo (63). More recently, the phase III LATITUDE trial has shown that the combination of Abiraterone plus prednisone with ADT conferred significant progression-free and overall survival benefits for patients with newly diagnosed high-risk metastatic castration-sensitive prostate cancer (64, 65).

### Repeated Resistance and Underlying Mechanisms

Unfortunately, while second-generation antigens can prolong the survival of castration-resistant prostate cancer patients, the relief is temporary. Once again, castration-resistant cancers become resistant to the newest inhibitors. The novel mutation F876L, which is evolved in the androgen receptor ligand binding domain during the treatment of enzalutamide, converts enzalutamide to an agonist (66–68). Enzalutamide-resistant prostate cancer can also bypass androgen receptor blockade by glucocorticoid receptor activation (69). Because the DNA binding domains of glucocorticoid receptor and androgen receptor are highly homologous and recognize identical DNA response elements, the glucocorticoid receptor can substitute for the androgen receptor to activate a subset of androgen receptor target genes which are required for prostate cancer survival and growth. In addition, the androgen receptor variant AR-V7 is associated with resistance to enzalutamide and Abiraterone (70, 71). Niclosamide, a novel inhibitor of AR-V7, may be able to overcome enzalutamide resistance (72). The crucial steroidogenic enzyme AKR1C3 is found to be overexpressed in enzalutamide-resistant prostate cancer cells and mediates enzalutamide resistance (73). The chemokine receptor CXCR7 is found to be overexpressed in enzalutamide-resistant prostate cancer cells and can activate MAPK to confer enzalutamide resistance (74). Up-regulation of coactivator GREB1 may also contribute to enzalutamide resistance (75).

One interesting observation is a reciprocal negative feedback regulation between AR and PI3K/Akt signaling pathways in prostate cancer. Pten loss contributed to the development of castration-resistant prostate cancer in mouse models (76, 77). It was thus postulated that combined inhibition of AR and PI3K pathways may achieve more potent inhibition of tumor growth. Indeed, in a phase Ib/II clinical trial, combination of abiraterone with Ipatasertib, an Akt inhibitor, showed more potent anticancer activity than abiraterone alone in metastatic castration-resistant prostate cancer patients (78). Moreover, the combination of Akt inhibitor AZD5363 and enzalutamide showed synergistic anti-prostate cancer effects in preclinical models (79) and has been tested in a phase I clinical trial (80). However, in another phase II clinical trial, a pan-class I PI3 kinase inhibitor BKM120 (buparlisib), with or without enzalutamide co-treatment, had only limited efficacy in men with metastatic castration-resistant prostate cancer (81).

## AR Co-factors in Prostate Cancer

Eukaryotic DNA wraps around histone proteins and forms an inhibitory chromatin structure. Gene activation by the androgen receptor requires assistance from other transcription factors. Among these factors, GATA2 and FoxA1 play particularly essential roles in androgen receptor signaling in prostate cancer cells. GATA2 belongs to the GATA family of transcription factors which contains six members in mammals. GATA2 factors bind to a consensus DNA sequence (A/T)GATA(A/G) and regulate gene expression. GATA factors are expressed in a tissue-specific manner and play fundamental roles in cell-fate specification (82). The role of GATA2 in androgen signaling was first implicated by the involvement of GATA2 in androgen regulation of the PSA gene (83). Binding motifs for GATA factors and Oct1 are enriched on AR binding regions in LNCaP cells, suggesting that these transcription factors cooperate with AR in mediating the androgen response (84). In addition to its co-factor function, GATA2 might directly regulate androgen receptor mRNA and protein expression in prostate cancer cells (85–87). Inhibition of GATA2 by small-molecule compounds is a potential strategy in blocking AR expression and signaling in castration-resistant prostate cancer (86).

FoxA1 is member of the forkhead family of DNA binding factors and plays a key role in androgen receptor-induced gene transcription. FoxA1 functions as a pioneer factor because it can bind to highly compacted chromatin and allows these genomic regions to be more accessible to other transcription factors. Therefore, FoxA1 functions to guide androgen receptor binding to the genomic sites in prostate cancer cells (88, 89). In normal prostate luminal epithelial cells, it plays an important role in maintaining the differentiation status. FoxA1 mutations occur frequently in primary and metastatic prostate cancers and may contribute to prostate tumorigenesis and cancer progression (90, 91). Loss of FoxA1 promotes prostate cancer progression to neuroendocrine small-cell prostate cancer (92). FoxA1 also has androgen receptor-independent function in prostate cancer (93).

## Novel Strategies in Castration-Resistant Prostate Cancer Treatments

Even with the latest androgen deprivation therapies, castration-resistant prostate cancers are rarely cured. They simply become resistant again. Strikingly, a substantial subset of these resistant cancers still express androgen receptors and/or their variants; their growth and survival are still dependent on androgen receptor signaling. Scientists in the field of prostate cancer research are relentless in pursuing novel strategies for more complete ablation of androgen receptor signaling.

Prompted by the clinical success of selective estrogen receptor downregulator (SERD) Faslodex (ICI 182,780 or Fulvestrant) (94), selective androgen receptor downregulators (SARDs) have been developed. For instance, a SARD compound AZD3514 (95) had undergone phase I clinical trial (96). Binding of SERD or SARD causes severe receptor conformational change, resulting in receptor degradation. Another strategy is to specifically degrade the androgen receptor protein through Proteolysis Targeting Chimeras (PROTACs). Briefly, a PROTAC molecule consists of two covalently linked ligands: one ligand binds to the target protein whereas the second ligand binds to an E3 ligase system. Several AR targeting PROTACs have been reported, including enzalutamide-derived ARCC-4 (97) and aryloxy tetramethylcyclobutane-derived ARD-69 (98, 99). ARCC-4 and ARD-69 represent a novel class of drugs which directly targets the androgen receptor protein for degradation, but their *in vivo* anti-prostate cancer activities remains to be established in mouse models. Similarly, small-molecule degraders of the Bromodomain and Extra-Terminal (BET) family of epigenetic regulators, which are essential for prostate cancer growth, showed *in vivo* anti-cancer efficacy in a castration-resistant VCaP xenograft mouse model (100).

Another strategy is to silence androgen receptor gene expression at the transcriptional level. Androgen receptor gene expression is driven by an orphan nuclear receptor RORγ in metastatic castration-resistant prostate cancer (101). RORγ antagonists XY018 and SR2211 potently suppressed the expression of the full length androgen receptor and truncated androgen receptor variants at the transcriptional level, consequently inhibiting prostate cancer growth in xenograft mouse model (101). It has been shown that enzalutamide-resistant prostate tumors are sensitive to RORγ antagonists, suggesting that such a strategy may be able to overcome resistance to second-generation antiandrogens. In comparison to older strategies, this treatment can silence the expression of both full length and truncated variant androgen receptors.

One more exciting area for cancer drug development is the use of synthetic lethality. Because a subset of cancers contains defects in their DNA repair system, they become particularly vulnerable to inhibition of DNA repair enzymes. Olaparib, an inhibitor of poly(ADP-ribose) polymerase (PARP) 1 and 2, two key enzymes involved in DNA repair, has been approved by FDA for germline BRCA-mutated metastatic breast cancer (102). In a phase II trial, olaparib produced a high response rate in castration-resistant prostate cancers with DNA-repair defects including BRCA2 loss and ATM aberrations (103). A phase II trial further shows that olaparib in combination with abiraterone increased progression-free survival in men with metastatic castration-resistant prostate cancer (104).

## Future Perspective

**Figure 3:**
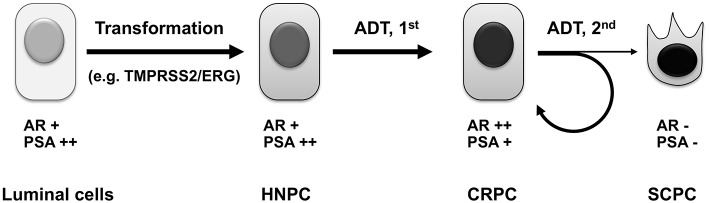
Evolution of prostate cancer under androgen deprivation therapy. Androgen sensitive primary prostate cancers arise from prostate luminal epithelial cells, which have undergone genetic alterations, such as mutation of PTEN tumor suppressor (107) or chromosomal rearrangement resulting in the TMPRSS2/ERG chimeric gene (108). Upon androgen deprivation including castration and the first-generation antiandrogen treatment, most HNPC will develop into CRPC, whose survival and growth still depends on androgen receptor signaling. After treatment with more potent androgen deprivation therapies such as second-generation antiandrogens, the majority of CRPC manages to develop novel mechanisms to maintain active androgen signaling axis to confer resistance, whereas a subset of CRPC will irreversibly lose androgen receptor expression, undergo divergent clonal evolution or de-differentiation, and become truly androgen-independent small-cell prostate cancer. ADT, androgen deprivation therapy; HNPC, hormone-naïve prostate cancer; CRPC, castration-resistant prostate cancer; SCPC, small-cell prostate cancer.

Although androgen deprivation therapy prolongs the survival and improves the quality of life of prostate cancer patients, it does not cure the disease. With more complete androgen deprivation therapies and androgen receptor ablation in the near future, we hypothesize that more castration-resistant prostate cancers will undergo de-differentiation, eventually lose androgen receptor expression, and become truly androgen-independent androgen receptor-negative small-cell prostate cancers (105, 106) (Figure 3). These small-cell prostate cancers have neuroendocrine markers or basal stem cell gene signatures (109), and they will no longer respond to hormone therapy or androgen receptor targeting therapy. There will be an urgent need to develop novel targeted therapies for this subtype of prostate cancer, when more small-cell prostate cancers are induced by complete androgen deprivation and androgen receptor ablation. It has been reported that these cancers contain gene amplification of AURKA and MYCN, which are promising therapeutic targets for this subtype of cancer (106).

Recent advances in immunotherapy are revolutionizing the treatment of cancer. For example, Sipuleucel-T (Provenge) for CRPC is the first FDA-approved therapeutic cancer vaccine (110). However, while the use of Sipuleucel-T prolonged overall survival, it did not lead to PSA reduction, tumor shrinkage, or improve disease free survival. The checkpoint blockade therapies using antibodies to block CTLA-4 or PD-1 have achieved long-term clinical benefits, and even cures a subset of cancers (111). Tumor infiltrating lymphocytes (TIL) have also shown huge promise in treating cancers (112). The success of checkpoint blockade and TIL therapies are dependent on the tumor mutational burden (113, 114). With more somatic mutations, cancer cells are more likely to be recognized by T lymphocytes as “non-self” foreigners and thereby likely to be eliminated by the immune system. Prostate cancer cells are known to have low mutation rates (115, 116) and therefore the vast majority of prostate cancers are insensitive to current single checkpoint blockade immunotherapies. Only a small subset of prostate cancers with mismatch repair defects or CDK12 mutations are likely to respond to checkpoint blockade (117, 118). Nevertheless, the combination of PD-1 and CTLA4 inhibitors in a phase II CheckMate 650 trial elicited durable clinical responses in metastatic castration-resistant prostate cancers (119). It is also possible that continuous androgen deprivation therapies will cause more mutations and genomic alternations, and render prostate cancer cells more vulnerable to immunotherapy (117).

In addition, the recently emerging chimeric antigen receptor (CAR) T cell therapy is a promising strategy for treatment of castration-resistant prostate cancer. The CAR T cell immunotherapy has recently been approved by FDA for treatment of refractory pre-B cell acute lymphoblastic leukemia and diffuse large B cell lymphoma (120). Because CAR-engineered T lymphocytes recognize cancer cells through cancer cell surface antigens, their anti-cancer activity is not dependent on mutations in cancer cells. This is particularly important for prostate cancers which harbor low amount of somatic mutations. In the literature, there are several reports of PSMA-specific CAR T-cell therapies which have shown anti-prostate cancer activity *in vitro* and in mouse models (121, 122). Additionally, CAR-engineered natural killer (NK) cell therapy is another promising treatment for castration-resistant prostate cancer. Taken together, with these new targeted and immune therapies in sight, scientists and patients can be optimistic about eventually winning the battle against castration-resistant prostate cancer.

## Author Contributions

QF and BH conceived and wrote the manuscript.

### Conflict of Interest Statement

The authors declare that the research was conducted in the absence of any commercial or financial relationships that could be construed as a potential conflict of interest.

## References

[1] He B Bowen NT Minges JT Wilson EM. Androgen-induced NH2- and COOH-terminal interaction inhibits p160 coactivator recruitment by activation function 2. J Biol Chem. (2001) 276:42293–301. 10.1074/jbc.M107492200 11551963

[2] Shaffer PL Jivan A Dollins DE Claessens F Gewirth DT. Structural basis of androgen receptor binding to selective androgen response elements. Proc Natl Acad Sci USA. (2004) 101:4758–63. 10.1073/pnas.0401123101 15037741 PMC387321

[3] Nadal M Prekovic S Gallastegui N Helsen C Abella M Zielinska K. Structure of the homodimeric androgen receptor ligand-binding domain. Nat Commun. (2017) 8:14388. 10.1038/ncomms14388 28165461 PMC5303882

[4] He B Kemppainen JA Wilson EM. FXXLF and WXXLF sequences mediate the NH2-terminal interaction with the ligand binding domain of the androgen receptor. J Biol Chem. (2000) 275:22986–94. 10.1074/jbc.M002807200 10816582

[5] He B Minges JT Lee LW Wilson EM. The FXXLF motif mediates androgen receptor-specific interactions with coregulators. J Biol Chem. (2002) 277:10226–35. 10.1074/jbc.M111975200 11779876

[6] He B Gampe RT Jr Kole AJ Hnat AT Stanley TB An G. Structural basis for androgen receptor interdomain and coactivator interactions suggests a transition in nuclear receptor activation function dominance. Mol Cell. (2004) 16:425–38. 10.1016/j.molcel.2004.09.036 15525515

[7] He B Lee LW Minges JT Wilson EM. Dependence of selective gene activation on the androgen receptor NH2- and COOH-terminal interaction. J Biol Chem. (2002) 277:25631–9. 10.1074/jbc.M202809200 12000757

[8] Heinlein CA Chang C. Androgen receptor (AR) coregulators: an overview. Endocr Rev. (2002) 23:175–200. 10.1210/edrv.23.2.0460 11943742

[9] He B Kemppainen JA Voegel JJ Gronemeyer H Wilson EM. Activation function 2 in the human androgen receptor ligand binding domain mediates interdomain communication with the NH(2)-terminal domain. J Biol Chem. (1999) 274:37219–25. 10.1074/jbc.274.52.37219 10601285

[10] Bai S He B Wilson EM. Melanoma antigen gene protein MAGE-11 regulates androgen receptor function by modulating the interdomain interaction. Mol Cell Biol. (2005) 25:1238–57. 10.1128/MCB.25.4.1238-1257.2005 15684378 PMC548016

[11] Wilson EM French FS. Binding properties of androgen receptors. Evidence for identical receptors in rat testis, epididymis, and prostate. J Biol Chem. (1976) 251:5620–9. 184085

[12] Grino PB Griffin JE Wilson JD. Testosterone at high concentrations interacts with the human androgen receptor similarly to dihydrotestosterone. Endocrinology. (1990) 126:1165–72. 10.1210/endo-126-2-1165 2298157

[13] Swinney DC. The role of binding kinetics in therapeutically useful drug action. Curr Opin Drug Discov Devel. (2009) 12:31–9. 19152211

[14] Huggins C Hodges CV Studies on prostatic cancer. I. The effect of castration, of estrogen and androgen injection on serum phosphatases in metastatic carcinoma of the prostate. Cancer Res. (1941) 1:293–7. 10.3322/canjclin.22.4.232 4625049

[15] Hellerstedt BA Pienta KJ. The current state of hormonal therapy for prostate cancer. CA Cancer J Clin. (2002) 52:154–79. 10.3322/canjclin.52.3.154 12018929

[16] de Voogt HJ. The position of cyproterone acetate (CPA), a steroidal anti-androgen, in the treatment of prostate cancer. Prostate Suppl. (1992) 4:91–5. 10.1002/pros.2990210514 1533452

[17] Simard J Luthy I Guay J Belanger A Labrie F. Characteristics of interaction of the antiandrogen flutamide with the androgen receptor in various target tissues. Mol Cell Endocrinol. (1986) 44:261–70. 10.1016/0303-7207(86)90132-2 3956856

[18] Kolvenbag GJ Furr BJ Blackledge GR. Receptor affinity and potency of non-steroidal antiandrogens: translation of preclinical findings into clinical activity. Prostate Cancer Prostatic Dis. (1998) 1:307–14. 10.1038/sj.pcan.4500262 12496872

[19] Joyce R Fenton MA Rode P Constantine M Gaynes L Kolvenbag G. High dose bicalutamide for androgen independent prostate cancer: effect of prior hormonal therapy. J Urol. (1998) 159:149–53. 10.1016/S0022-5347(01)64039-4 9400459

[20] Labrie F Dupont A Belanger A Cusan L Lacourciere Y Monfette G. New hormonal therapy in prostatic carcinoma: combined treatment with an LHRH agonist and an antiandrogen. Clin Invest Med. (1982) 5:267–75. 6819101

[21] Labrie F Dupont A Belanger A St-Arnaud R Giguere M Lacourciere Y. Treatment of prostate cancer with gonadotropin-releasing hormone agonists. Endocr Rev. (1986) 7:67–74. 10.1210/edrv-7-1-67 3514203

[22] Dijkman GA Janknegt RA De Reijke TM Debruyne FM. Long-term efficacy and safety of nilutamide plus castration in advanced prostate cancer, and the significance of early prostate specific antigen normalization. International Anandron Study Group. J Urol. (1997) 158:160–3. 10.1097/00005392-199707000-00051 9186345

[23] Maximum androgen blockade in advanced prostate cancer: an overview of the randomised trials. Prostate Cancer Trialists' Collaborative Group. Lancet. (2000) 355:1491–8. 10.1016/S0140-6736(00)02163-2 10801170

[24] Mohler JL Gregory CW Ford OH III Kim D Weaver CM Petrusz P. The androgen axis in recurrent prostate cancer. Clin Cancer Res. (2004) 10:440–8. 10.1158/1078-0432.CCR-1146-03 14760063

[25] Titus MA Schell MJ Lih FB Tomer KB Mohler JL. Testosterone and dihydrotestosterone tissue levels in recurrent prostate cancer. Clin Cancer Res. (2005) 11:4653–7. 10.1158/1078-0432.CCR-05-0525 16000557

[26] Montgomery RB Mostaghel EA Vessella R Hess DL Kalhorn TF Higano CS. Maintenance of intratumoral androgens in metastatic prostate cancer: a mechanism for castration-resistant tumor growth. Cancer Res. (2008) 68:4447–54. 10.1158/0008-5472.CAN-08-0249 18519708 PMC2536685

[27] Feldman BJ Feldman D. The development of androgen-independent prostate cancer. Nat Rev Cancer. (2001) 1:34–45. 10.1038/35094009 11900250

[28] Scher HI Buchanan G Gerald W Butler LM Tilley WD. Targeting the androgen receptor: improving outcomes for castration-resistant prostate cancer. Endocr Relat Cancer. (2004) 11:459–76. 10.1677/erc.1.00525 15369448

[29] Visakorpi T Hyytinen E Koivisto P Tanner M Keinanen R Palmberg C. *In vivo* amplification of the androgen receptor gene and progression of human prostate cancer. Nat Genet. (1995) 9:401–6. 10.1038/ng0495-401 7795646

[30] Linja MJ Savinainen KJ Saramaki OR Tammela TL Vessella RL Visakorpi T. Amplification and overexpression of androgen receptor gene in hormone-refractory prostate cancer. Cancer Res. (2001) 61:3550–5. 11325816

[31] Koivisto P Kononen J Palmberg C Tammela T Hyytinen E Isola J. Androgen receptor gene amplification: a possible molecular mechanism for androgen deprivation therapy failure in prostate cancer. Cancer Res. (1997) 57:314–9. 9000575

[32] Chen CD Welsbie DS Tran C Baek SH Chen R Vessella R. Molecular determinants of resistance to antiandrogen therapy. Nat Med. (2004) 10:33–9. 10.1038/nm972 14702632

[33] Robinson D Van Allen EM Wu YM Schultz N Lonigro RJ Mosquera JM. Integrative clinical genomics of advanced prostate cancer. Cell. (2015) 161:1215–28. 10.1016/j.cell.2015.06.053 26000489 PMC4484602

[34] Takeda DY Spisak S Seo JH Bell C O'Connor E Korthauer K. A somatically acquired enhancer of the androgen receptor is a noncoding driver in advanced prostate cancer. Cell. (2018) 174:422–32.e13. 10.1016/j.cell.2018.05.037 29909987 PMC6046260

[35] Quigley DA Dang HX Zhao SG Lloyd P Aggarwal R Alumkal JJ. Genomic hallmarks and structural variation in metastatic prostate cancer. Cell. (2018) 174:758–69.e9. 10.1016/j.cell.2018.06.039 30033370 PMC6425931

[36] Gregory CW He B Johnson RT Ford OH Mohler JL French FS. A mechanism for androgen receptor-mediated prostate cancer recurrence after androgen deprivation therapy. Cancer Res. (2001) 61:4315–9. 11389051

[37] Karpf AR Bai S James SR Mohler JL Wilson EM. Increased expression of androgen receptor coregulator MAGE-11 in prostate cancer by DNA hypomethylation and cyclic AMP. Mol Cancer Res. (2009) 7:523–35. 10.1158/1541-7786.MCR-08-0400 19372581 PMC2670465

[38] Minges JT Su S Grossman G Blackwelder AJ Pop EA Mohler JL. Melanoma antigen-A11 (MAGE-A11) enhances transcriptional activity by linking androgen receptor dimers. J Biol Chem. (2013) 288:1939–52. 10.1074/jbc.M112.428409 23172223 PMC3548502

[39] Tilley WD Buchanan G Hickey TE Bentel JM. Mutations in the androgen receptor gene are associated with progression of human prostate cancer to androgen independence. Clin Cancer Res. (1996) 2:277–85. 9816170

[40] Veldscholte J Ris-Stalpers C Kuiper GG Jenster G Berrevoets C Claassen E. A mutation in the ligand binding domain of the androgen receptor of human LNCaP cells affects steroid binding characteristics and response to anti-androgens. Biochem Biophys Res Commun. (1990) 173:534–40. 10.1016/S0006-291X(05)80067-1 2260966

[41] Taplin ME Bubley GJ Shuster TD Frantz ME Spooner AE Ogata GK. Mutation of the androgen-receptor gene in metastatic androgen-independent prostate cancer. N Engl J Med. (1995) 332:1393–8. 10.1056/NEJM199505253322101 7723794

[42] Zhao XY Malloy PJ Krishnan AV Swami S Navone NM Peehl DM. Glucocorticoids can promote androgen-independent growth of prostate cancer cells through a mutated androgen receptor. Nat Med. (2000) 6:703–6. 10.1038/76287 10835690

[43] Hu R Dunn TA Wei S Isharwal S Veltri RW Humphreys E. Ligand-independent androgen receptor variants derived from splicing of cryptic exons signify hormone-refractory prostate cancer. Cancer Res. (2009) 69:16–22. 10.1158/0008-5472.CAN-08-2764 19117982 PMC2614301

[44] Guo Z Yang X Sun F Jiang R Linn DE Chen H. A novel androgen receptor splice variant is up-regulated during prostate cancer progression and promotes androgen depletion-resistant growth. Cancer Res. (2009) 69:2305–13. 10.1158/0008-5472.CAN-08-3795 19244107 PMC2672822

[45] Dehm SM Schmidt LJ Heemers HV Vessella RL Tindall DJ. Splicing of a novel androgen receptor exon generates a constitutively active androgen receptor that mediates prostate cancer therapy resistance. Cancer Res. (2008) 68:5469–77. 10.1158/0008-5472.CAN-08-0594 18593950 PMC2663383

[46] Gregory CW Fei X Ponguta LA He B Bill HM French FS. Epidermal growth factor increases coactivation of the androgen receptor in recurrent prostate cancer. J Biol Chem. (2004) 279:7119–30. 10.1074/jbc.M307649200 14662770

[47] Culig Z Hobisch A Cronauer MV Radmayr C Trapman J Hittmair A. Androgen receptor activation in prostatic tumor cell lines by insulin-like growth factor-I, keratinocyte growth factor, and epidermal growth factor. Cancer Res. (1994) 54:5474–8. 7522959

[48] Kelly WK Scher HI. Prostate specific antigen decline after antiandrogen withdrawal: the flutamide withdrawal syndrome. J Urol. (1993) 149:607–9. 10.1016/S0022-5347(17)36163-3 7679759

[49] Small EJ Carroll PR. Prostate-specific antigen decline after casodex withdrawal: evidence for an antiandrogen withdrawal syndrome. Urology. (1994) 43:408–10. 10.1016/0090-4295(94)90092-2 7510915

[50] Huan SD Gerridzen RG Yau JC Stewart DJ. Antiandrogen withdrawal syndrome with nilutamide. Urology. (1997) 49:632–4. 10.1016/S0090-4295(96)00558-4 9111642

[51] Fenton MA Shuster TD Fertig AM Taplin ME Kolvenbag G Bubley GJ. Functional characterization of mutant androgen receptors from androgen-independent prostate cancer. Clin Cancer Res. (1997) 3:1383–8. 9815822

[52] Tran C Ouk S Clegg NJ Chen Y Watson PA Arora V. Development of a second-generation antiandrogen for treatment of advanced prostate cancer. Science. (2009) 324:787–90. 10.1126/science.1168175 19359544 PMC2981508

[53] Moilanen AM Riikonen R Oksala R Ravanti L Aho E Wohlfahrt G. Discovery of ODM-201, a new-generation androgen receptor inhibitor targeting resistance mechanisms to androgen signaling-directed prostate cancer therapies. Sci Rep. (2015) 5:12007. 10.1038/srep12007 26137992 PMC4490394

[54] Rathkopf DE Saleh MN Tsai FYC Rosen LS Adams BJ Liu L An open-label phase 1/2a study to evaluate the safety, pharmacokinetics, pharmacodynamics, and preliminary efficacy of TRC253, an androgen receptor antagonist, in patients with metastatic castration-resistant prostate cancer (mCRPC). J Clin Oncol. (2018) 36:TPS403 10.1200/JCO.2018.36.6_suppl.TPS403

[55] Scher HI Fizazi K Saad F Taplin ME Sternberg CN Miller K. Increased survival with enzalutamide in prostate cancer after chemotherapy. N Engl J Med. (2012) 367:1187–97. 10.1056/NEJMoa1207506 22894553

[56] Beer TM Armstrong AJ Rathkopf DE Loriot Y Sternberg CN Higano CS Enzalutamide in metastatic prostate cancer before chemotherapy. N Engl J Med. (2014) 371:424–33. 10.1056/NEJMoa1405095 24881730 PMC4418931

[57] Smith MR Saad F Chowdhury S Oudard S Hadaschik BA Graff JN. Apalutamide treatment and metastasis-free survival in prostate cancer. N Engl J Med. (2018) 378:1408–18. 10.1056/NEJMoa1715546 29420164

[58] Hussain M Fizazi K Saad F Rathenborg P Shore N Ferreira U. Enzalutamide in men with nonmetastatic, castration-resistant prostate cancer. N Engl J Med. (2018) 378:2465–74. 10.1056/NEJMoa1800536 29949494 PMC8288034

[59] Fizazi K Shore N Tammela TL Ulys A Vjaters E Polyakov S. Darolutamide in nonmetastatic, castration-resistant prostate cancer. N Engl J Med. (2019) 380:1235–46. 10.1056/NEJMoa1815671 30763142

[60] Chi KN Agarwal N Bjartell A Chung BH Pereira de Santana Gomes AJ Given R. Apalutamide for metastatic, castration-sensitive prostate cancer. N Engl J Med. (2019) 381:13–24. 10.1056/NEJMoa1903307 31150574

[61] Davis ID Martin AJ Stockler MR Begbie S Chi KN Chowdhury S. Enzalutamide with standard first-line therapy in metastatic prostate cancer. N Engl J Med. (2019) 381:121–31. 10.1056/NEJMoa1903835 31157964

[62] Armstrong AJ Szmulewitz RZ Petrylak DP Holzbeierlein J Villers A Azad A. ARCHES: A randomized, phase III study of androgen deprivation therapy with enzalutamide or placebo in men with metastatic hormone-sensitive prostate cancer. J Clin Oncol. (2019). 10.1200/JCO.19.00799. [Epub ahead of print]. 31329516 PMC6839905

[63] de Bono JS Logothetis CJ Molina A Fizazi K North S Chu L. Abiraterone and increased survival in metastatic prostate cancer. N Engl J Med. (2011) 364:1995–2005. 10.1056/NEJMoa1014618 21612468 PMC3471149

[64] Fizazi K Tran N Fein L Matsubara N Rodriguez-Antolin A Alekseev BY. Abiraterone acetate plus prednisone in patients with newly diagnosed high-risk metastatic castration-sensitive prostate cancer (LATITUDE): final overall survival analysis of a randomised, double-blind, phase 3 trial. Lancet Oncol. (2019) 20:686–700. 10.1016/S1470-2045(19)30082-8 30987939

[65] Fizazi K Tran N Fein L Matsubara N Rodriguez-Antolin A Alekseev BY. Abiraterone plus prednisone in metastatic, castration-sensitive prostate cancer. N Engl J Med. (2017) 377:352–60. 10.1056/NEJMoa1704174 28578607

[66] Korpal M Korn JM Gao X Rakiec DP Ruddy DA Doshi S. An F876L mutation in androgen receptor confers genetic and phenotypic resistance to MDV3100 (enzalutamide). Cancer Discov. (2013) 3:1030–43. 10.1158/2159-8290.CD-13-0142 23842682

[67] Joseph JD Lu N Qian J Sensintaffar J Shao G Brigham D. A clinically relevant androgen receptor mutation confers resistance to second-generation antiandrogens enzalutamide and ARN-509. Cancer Discov. (2013) 3:1020–9. 10.1158/2159-8290.CD-13-0226 23779130

[68] Lallous N Volik SV Awrey S Leblanc E Tse R Murillo J. Functional analysis of androgen receptor mutations that confer anti-androgen resistance identified in circulating cell-free DNA from prostate cancer patients. Genome Biol. (2016) 17:10. 10.1186/s13059-015-0864-1 26813233 PMC4729137

[69] Arora VK Schenkein E Murali R Subudhi SK Wongvipat J Balbas MD. Glucocorticoid receptor confers resistance to antiandrogens by bypassing androgen receptor blockade. Cell. (2013) 155:1309–22. 10.1016/j.cell.2013.11.012 24315100 PMC3932525

[70] Antonarakis ES Lu C Wang H Luber B Nakazawa M Roeser JC. AR-V7 and resistance to enzalutamide and abiraterone in prostate cancer. N Engl J Med. (2014) 371:1028–38. 10.1056/NEJMoa1315815 25184630 PMC4201502

[71] Li Y Chan SC Brand LJ Hwang TH Silverstein KA Dehm SM. Androgen receptor splice variants mediate enzalutamide resistance in castration-resistant prostate cancer cell lines. Cancer Res. (2013) 73:483–9. 10.1158/0008-5472.CAN-12-3630 23117885 PMC3549016

[72] Liu C Lou W Zhu Y Nadiminty N Schwartz CT Evans CP. Niclosamide inhibits androgen receptor variants expression and overcomes enzalutamide resistance in castration-resistant prostate cancer. Clin Cancer Res. (2014) 20:3198–210. 10.1158/1078-0432.CCR-13-3296 24740322 PMC4058390

[73] Liu C Lou W Zhu Y Yang JC Nadiminty N Gaikwad NW. Intracrine androgens and AKR1C3 activation confer resistance to enzalutamide in prostate cancer. Cancer Res. (2015) 75:1413–22. 10.1158/0008-5472.CAN-14-3080 25649766 PMC4383695

[74] Li S Fong KW Gritsina G Zhang A Zhao JC Kim J. Activation of MAPK signaling by CXCR7 leads to enzalutamide resistance in prostate cancer. Cancer Res. (2019) 79:2580–92. 10.1158/0008-5472.CAN-18-2812 30952632 PMC6522281

[75] Lee E Wongvipat J Choi D Wang P Lee YS Zheng D. GREB1 amplifies androgen receptor output in human prostate cancer and contributes to antiandrogen resistance. Elife. (2019) 8:e41913. 10.7554/eLife.41913 30644358 PMC6336405

[76] Carver BS Chapinski C Wongvipat J Hieronymus H Chen Y Chandarlapaty S. Reciprocal feedback regulation of PI3K and androgen receptor signaling in PTEN-deficient prostate cancer. Cancer Cell. (2011) 19:575–86. 10.1016/j.ccr.2011.04.008 21575859 PMC3142785

[77] Mulholland DJ Tran LM Li Y Cai H Morim A Wang S. Cell autonomous role of PTEN in regulating castration-resistant prostate cancer growth. Cancer Cell. (2011) 19:792–804. 10.1016/j.ccr.2011.05.006 21620777 PMC3157296

[78] de Bono JS De Giorgi U Rodrigues DN Massard C Bracarda S Font A. Randomized phase II study evaluating Akt blockade with Ipatasertib, in combination with abiraterone, in patients with metastatic prostate cancer with and without PTEN loss. Clin Cancer Res. (2019) 25:928–36. 10.1158/1078-0432.CCR-18-0981 30037818

[79] Toren P Kim S Cordonnier T Crafter C Davies BR Fazli L. Combination AZD5363 with enzalutamide significantly delays enzalutamide-resistant prostate cancer in preclinical models. Eur Urol. (2015) 67:986–90. 10.1016/j.eururo.2014.08.006 25151012

[80] Kolinsky MP Rescigno P Bianchini D Zafeiriou Z Mehra N Mateo J A phase I dose-escalation study of enzalutamide in combination with the AKT inhibitor AZD5363 in patients with mCRPC. J Clin Oncol. (2017) 35:135 10.1200/JCO.2017.35.6_suppl.135 32205016 PMC7217345

[81] Armstrong AJ Halabi S Healy P Alumkal JJ Winters C Kephart J Phase II trial of the PI3 kinase inhibitor buparlisib (BKM-120) with or without enzalutamide in men with metastatic castration resistant prostate cancer. Eur J Cancer. (2017) 81:228–36. 10.1016/j.ejca.2017.02.030 28502694 PMC6345641

[82] Patient RK McGhee JD. The GATA family (vertebrates and invertebrates). Curr Opin Genet Dev. (2002) 12:416–22. 10.1016/S0959-437X(02)00319-2 12100886

[83] Perez-Stable CM Pozas A Roos BA. A role for GATA transcription factors in the androgen regulation of the prostate-specific antigen gene enhancer. Mol Cell Endocrinol. (2000) 167:43–53. 10.1016/S0303-7207(00)00300-2 11000519

[84] Wang Q Li W Liu XS Carroll JS Janne OA Keeton EK. A hierarchical network of transcription factors governs androgen receptor-dependent prostate cancer growth. Mol Cell. (2007) 27:380–92. 10.1016/j.molcel.2007.05.041 17679089 PMC3947890

[85] Bohm M Locke WJ Sutherland RL Kench JG Henshall SM. A role for GATA-2 in transition to an aggressive phenotype in prostate cancer through modulation of key androgen-regulated genes. Oncogene. (2009) 28:3847–56. 10.1038/onc.2009.243 19684615

[86] He B Lanz RB Fiskus W Geng C Yi P Hartig SM. GATA2 facilitates steroid receptor coactivator recruitment to the androgen receptor complex. Proc Natl Acad Sci USA. (2014) 111:18261–6. 10.1073/pnas.1421415111 25489091 PMC4280633

[87] Wu D Sunkel B Chen Z Liu X Ye Z Li Q. Three-tiered role of the pioneer factor GATA2 in promoting androgen-dependent gene expression in prostate cancer. Nucleic Acids Res. (2014) 42:3607–22. 10.1093/nar/gkt1382 24423874 PMC3973339

[88] Sahu B Laakso M Pihlajamaa P Ovaska K Sinielnikov I Hautaniemi S. FoxA1 specifies unique androgen and glucocorticoid receptor binding events in prostate cancer cells. Cancer Res. (2013) 73:1570–80. 10.1158/0008-5472.CAN-12-2350 23269278

[89] Sahu B Laakso M Ovaska K Mirtti T Lundin J Rannikko A. Dual role of FoxA1 in androgen receptor binding to chromatin, androgen signalling and prostate cancer. EMBO J. (2011) 30:3962–76. 10.1038/emboj.2011.328 21915096 PMC3209787

[90] Parolia A Cieslik M Chu SC Xiao L Ouchi T Zhang Y. Distinct structural classes of activating FOXA1 alterations in advanced prostate cancer. Nature. (2019). 571:413–8. 10.1158/1538-7445.SABCS18-4497 31243372 PMC6661908

[91] Adams EJ Karthaus WR Hoover E Liu D Gruet A Zhang Z. FOXA1 mutations alter pioneering activity, differentiation and prostate cancer phenotypes. Nature. (2019) 571:408–12. 10.1038/s41586-019-1318-9 31243370 PMC6661172

[92] Kim J Jin H Zhao JC Yang YA Li Y Yang X. FOXA1 inhibits prostate cancer neuroendocrine differentiation. Oncogene. (2017) 36:4072–80. 10.1038/onc.2017.50 28319070 PMC5509480

[93] Jin HJ Zhao JC Ogden I Bergan RC Yu J. Androgen receptor-independent function of FoxA1 in prostate cancer metastasis. Cancer Res. (2013) 73:3725–36. 10.1158/0008-5472.CAN-12-3468 23539448 PMC3686855

[94] Howell A Robertson JF Quaresma Albano J Aschermannova A Mauriac L Kleeberg UR. Fulvestrant, formerly ICI 182,780, is as effective as anastrozole in postmenopausal women with advanced breast cancer progressing after prior endocrine treatment. J Clin Oncol. (2002) 20:3396–403. 10.1200/JCO.2002.10.057 12177099

[95] Bradbury RH Acton DG Broadbent NL Brooks AN Carr GR Hatter G. Discovery of AZD3514, a small-molecule androgen receptor downregulator for treatment of advanced prostate cancer. Bioorg Med Chem Lett. (2013) 23:1945–8. 10.1016/j.bmcl.2013.02.056 23466225

[96] Omlin A Jones RJ van der Noll R Satoh T Niwakawa M Smith SA. AZD3514, an oral selective androgen receptor down-regulator in patients with castration-resistant prostate cancer - results of two parallel first-in-human phase I studies. Invest New Drugs. (2015) 33:679–90. 10.1007/s10637-015-0235-5 25920479

[97] Salami J Alabi S Willard RR Vitale NJ Wang J Dong H. Androgen receptor degradation by the proteolysis-targeting chimera ARCC-4 outperforms enzalutamide in cellular models of prostate cancer drug resistance. Commun Biol. (2018) 1:100. 10.1038/s42003-018-0105-8 30271980 PMC6123676

[98] Han X Wang C Qin C Xiang W Fernandez-Salas E Yang CY. Discovery of ARD-69 as a highly potent proteolysis targeting chimera (PROTAC) degrader of androgen receptor (AR) for the treatment of prostate cancer. J Med Chem. (2019) 62:941–64. 10.1021/acs.jmedchem.8b01631 30629437

[99] Guo C Linton A Kephart S Ornelas M Pairish M Gonzalez J. Discovery of aryloxy tetramethylcyclobutanes as novel androgen receptor antagonists. J Med Chem. (2011) 54:7693–704. 10.1021/jm201059s 21936524

[100] Kregel S Malik R Asangani IA Wilder-Romans K Rajendiran T Xiao L Functional and mechanistic interrogation of BET bromodomain degraders for the treatment of metastatic castration-resistant prostate cancer. Clin Cancer Res. (2019) 78(Suppl. 13):5795 10.1158/1538-7445.AM2018-5795 PMC6606381 30918020

[101] Wang J Zou JX Xue X Cai D Zhang Y Duan Z ROR-gamma drives androgen receptor expression and represents a therapeutic target in castration-resistant prostate cancer. Nat Med. (2016) 22:488–96. 10.1038/nm.4070 27019329 PMC5030109

[102] Robson M Im SA Senkus E Xu B Domchek SM Masuda N. Olaparib for metastatic breast cancer in patients with a germline BRCA mutation. N Engl J Med. (2017) 377:523–33. 10.1056/NEJMoa1706450 28578601

[103] Mateo J Carreira S Sandhu S Miranda S Mossop H Perez-Lopez R. DNA-repair defects and olaparib in metastatic prostate cancer. N Engl J Med. (2015) 373:1697–708. 10.1056/NEJMoa1506859 26510020 PMC5228595

[104] Clarke N Wiechno P Alekseev B Sala N Jones R Kocak I. Olaparib combined with abiraterone in patients with metastatic castration-resistant prostate cancer: a randomised, double-blind, placebo-controlled, phase 2 trial. Lancet Oncol. (2018) 19:975–86. 10.1016/S1470-2045(18)30365-6 29880291

[105] Beltran H Prandi D Mosquera JM Benelli M Puca L Cyrta J. Divergent clonal evolution of castration-resistant neuroendocrine prostate cancer. Nat Med. (2016) 22:298–305. 10.1038/nm.4045 26855148 PMC4777652

[106] Beltran H Rickman DS Park K Chae SS Sboner A MacDonald TY. Molecular characterization of neuroendocrine prostate cancer and identification of new drug targets. Cancer Discov. (2011) 1:487–95. 10.1158/2159-8290.CD-11-0130 22389870 PMC3290518

[107] Li J Yen C Liaw D Podsypanina K Bose S Wang SI. PTEN, a putative protein tyrosine phosphatase gene mutated in human brain, breast, and prostate cancer. Science. (1997) 275:1943–7. 10.1126/science.275.5308.1943 9072974

[108] Tomlins SA Rhodes DR Perner S Dhanasekaran SM Mehra R Sun XW. Recurrent fusion of TMPRSS2 and ETS transcription factor genes in prostate cancer. Science. (2005) 310:644–8. 10.1126/science.1117679 16254181

[109] Smith BA Sokolov A Uzunangelov V Baertsch R Newton Y Graim K. A basal stem cell signature identifies aggressive prostate cancer phenotypes. Proc Natl Acad Sci USA. (2015) 112:E6544–52. 10.1073/pnas.1518007112 26460041 PMC4664352

[110] Kantoff PW Higano CS Shore ND Berger ER Small EJ Penson DF. Sipuleucel-T immunotherapy for castration-resistant prostate cancer. N Engl J Med. (2010) 363:411–22. 10.1056/NEJMoa1001294 20818862

[111] Topalian SL Hodi FS Brahmer JR Gettinger SN Smith DC McDermott DF. Safety, activity, and immune correlates of anti-PD-1 antibody in cancer. N Engl J Med. (2012) 366:2443–54. 10.1056/NEJMoa1200690 22658127 PMC3544539

[112] Zacharakis N Chinnasamy H Black M Xu H Lu YC Zheng Z. Immune recognition of somatic mutations leading to complete durable regression in metastatic breast cancer. Nat Med. (2018) 24:724–30. 10.1038/s41591-018-0040-8 29867227 PMC6348479

[113] Goodman AM Kato S Bazhenova L Patel SP Frampton GM Miller V. Tumor mutational burden as an independent predictor of response to immunotherapy in diverse cancers. Mol Cancer Ther. (2017) 16:2598–608. 10.1158/1535-7163.MCT-17-0386 28835386 PMC5670009

[114] Hellmann MD Ciuleanu TE Pluzanski A Lee JS Otterson GA Audigier-Valette C. Nivolumab plus ipilimumab in lung cancer with a high tumor mutational burden. N Engl J Med. (2018) 378:2093–104. 10.1056/NEJMoa1801946 29658845 PMC7193684

[115] Alexandrov LB Nik-Zainal S Wedge DC Aparicio SA Behjati S Biankin AV. Signatures of mutational processes in human cancer. Nature. (2013) 500:415–21. 10.1038/nature12477 23945592 PMC3776390

[116] Martincorena I Campbell PJ. Somatic mutation in cancer and normal cells. Science. (2015) 349:1483–9. 10.1126/science.aab4082 26404825

[117] Wu YM Cieslik M Lonigro RJ Vats P Reimers MA Cao X. Inactivation of CDK12 delineates a distinct immunogenic class of advanced prostate cancer. Cell. (2018) 173:1770–82.e14. 10.1016/j.cell.2018.04.034 29906450 PMC6084431

[118] Le DT Durham JN Smith KN Wang H Bartlett BR Aulakh LK. Mismatch repair deficiency predicts response of solid tumors to PD-1 blockade. Science. (2017) 357:409–13. 10.1126/science.aan6733 28596308 PMC5576142

[119] Anti-PD-1-CTLA4 Combo hits prostate cancer. Cancer Discov. (2019) 9:569–70. 10.1158/2159-8290.CD-NB2019-039 30894362

[120] June CH O'Connor RS Kawalekar OU Ghassemi S Milone MC. CAR T cell immunotherapy for human cancer. Science. (2018) 359:1361–5. 10.1126/science.aar6711 29567707

[121] Kloss CC Lee J Zhang A Chen F Melenhorst JJ Lacey SF. Dominant-negative TGF-beta receptor enhances PSMA-targeted human CAR T cell proliferation and augments prostate cancer eradication. Mol Ther. (2018) 26:1855–66. 10.1016/j.ymthe.2018.05.003 29807781 PMC6037129

[122] Junghans RP Ma Q Rathore R Gomes EM Bais AJ Lo AS. Phase I trial of anti-PSMA designer CAR-T cells in prostate cancer: possible role for interacting interleukin 2-T cell pharmacodynamics as a determinant of clinical response. Prostate. (2016) 76:1257–70. 10.1002/pros.23214 27324746

